# Identification of potential biomarkers for idiopathic pulmonary arterial hypertension using single-cell and bulk RNA sequencing analysis

**DOI:** 10.3389/fgene.2024.1328234

**Published:** 2024-03-22

**Authors:** Yan Du, Jingqiu Zhang, Kai Guo, Yongxiang Yin

**Affiliations:** ^1^ Department of Pathology, Wuxi Maternity and Child Healthcare Hospital, Affiliated Women’s Hospital of Jiangnan University, Jiangnan University, Wuxi, Jiangsu, China; ^2^ Department of Pathology, Suzhou Science and Technology Town Hospital, Suzhou, China

**Keywords:** idiopathic pulmonary arterial hypertension, arterial endothelial cells, single cell RNA sequencing, bulk RNA sequencing, biomarkers

## Abstract

Idiopathic pulmonary arterial hypertension (IPAH) is a rare and severe cardiopulmonary disease with a challenging prognosis, and its underlying pathogenesis remains elusive. A comprehensive understanding of IPAH is crucial to unveil potential diagnostic markers and therapeutic targets. In this study, we investigated cellular heterogeneity and molecular pathology in IPAH using single-cell RNA sequencing (scRNA-seq) analysis. Our scRNA-seq results revealed significant alterations in three crucial signaling pathways in IPAH: the hypoxia pathway, TGF β pathway, and ROS pathway, primarily attributed to changes in gene expression within arterial endothelial cells. Moreover, through bulk RNA sequencing analysis, we identified differentially expressed genes (DEGs) enriched in GO and KEGG pathways, implicated in regulating cell adhesion and oxidative phosphorylation in IPAH lungs. Similarly, DEGs-enriched pathways in IPAH arterial endothelial cells were also identified. By integrating DEGs from three IPAH datasets and applying protein-protein interaction (PPI) analysis, we identified 12 candidate biomarkers. Subsequent validation in two additional PAH datasets led us to highlight five potential biomarkers (CTNNB1, MAPK3, ITGB1, HSP90AA1, and DDX5) with promising diagnostic significance for IPAH. Furthermore, real-time quantitative polymerase chain reaction (RT-qPCR) confirmed significant differences in the expression of these five genes in pulmonary arterial endothelial cells from PAH mice. In conclusion, our findings shed light on the pivotal role of arterial endothelial cells in the development of IPAH. Furthermore, the integration of single-cell and bulk RNA sequencing datasets allowed us to pinpoint novel candidate biomarkers for the diagnosis of IPAH. This work opens up new avenues for research and potential therapeutic interventions in IPAH management.

## 1 Introduction

Pulmonary arterial hypertension (PAH) is a rare syndrome characterized by dyspnea and fatigue resulting from progressive increases in pulmonary vascular resistance (PVR) and eventual right ventricular (RV) failure (Naeije et al., 2022). PAH is characterized by an elevation in mean pulmonary arterial pressure exceeding 20 mmHg from the normal range of 10–20 mmHg at rest, which is assessed through right heart catheterization (Evans et al., 2021). Idiopathic PAH (IPAH) constitutes approximately half of the cases and is characterized by an unexplained elevation in pulmonary vascular resistance, leading to a persistent increase in pulmonary arterial pressure at rest, with a minimum threshold of ≥20 mmHg, while excluding all secondary causes of pulmonary arterial hypertension (Runo and Loyd, 2003; Farber et al., 2015).

The pathophysiology of IPAH is intricate and largely unknown, with genetic factors considered to contribute to the alterations in vascular structure and function observed in PAH. While BMPR2 mutations have been identified in some cases, a second unidentified hit may modulate disease progression (Eichstaedt et al., 2016; Girerd et al., 2017). Therefore, understanding the potential permissive effect of common genetic variants on the development of IPAH becomes crucial.

Endothelial cells (ECs) play a crucial role in PAH pathogenesis, as they are known to be damaged and/or dysfunctional in patients with the condition (Cella et al., 2001; Wolff et al., 2007). Factors contributing to EC injury include hypoxia, inhibition of survival signaling, inflammatory cytokines, as well as pathological shear stress and fluid mechanics in the pulmonary circulation raised by left-to-right shunts in congenital heart disease (Hughes et al., 2005; Veith et al., 2022). Dysfunctional EC signaling is associated with various characteristics of PAH, including oxidative/nitrative stress, pulmonary inflammation, coagulation, metabolic shift, proliferation, and altered vascular cell viability (Legchenko et al., 2018; Jiang et al., 2021). Recent research employing single-cell RNA sequencing in PAH has revealed changes in ECs, including upregulation of major histocompatibility complex class II pathways and specific responses in capillary EC subpopulations, further supporting the role of ECs in the inflammatory response to PAH (Rodor et al., 2022). However, there remains insufficient understanding of EC heterogeneity in IPAH, highlighting the importance of identifying biomarkers for IPAH treatment through a combination of single-cell RNA sequencing and bulk RNA sequencing analysis.

The purpose of this study is to identify key biomarkers for the clinical diagnosis of IPAH by analyzing the cellular landscape and expression differences in patients with IPAH using single-cell RNA analysis.

## 2 Methods

### 2.1 Data download

The single-cell dataset GSE169471 was obtained from the GEO (https://www.ncbi.nlm.nih.gov/gds) database, which contained 3 samples from patients with IPAH and 6 samples from normal control lungs (Saygin et al., 2020). The bulk RNA-seq dataset GSE113439 containing 11 healthy tissues and 6 patients with IPAH was also downloaded (Mura et al., 2019). For the GSE185479 scRNA-seq dataset containing human pulmonary arterial endothelial cells from 3 health and 3 PAH lungs (Asosingh et al., 2021). The gene expression profile datasets GSE126262 (Reyes-Palomares et al., 2020) and GSE130391 (Halliday et al., 2020) were downloaded to identify biomarker genes. All datasets are presented in Supplementary Table S1.

### 2.2 scRNA sequencing data processing

For the scRNA-seq datasets, we first downloaded the raw data for quality control and data filtering (cells with <200 genes, >5,000 genes, or >10% mitochondrial genes) using the R package Seurat (version 4.2.2). After data normalization, 2000 highly variable genes (HVGs) were identified with “vst” method for each sample. Significant principal components (PCs) were identified through application of PCA, and the *p*-value distribution was visualized using the JackStraw and ScoreJackStraw functions. To mitigate the potential batch effect of sample identity that could interfere with downstream analysis, the R package Harmony (version 0.1.0) was utilized for batch correction (Korsunsky et al., 2019). Cell classification with a resolution of 0.5 was achieved using the FindClusters function. Differential expression analysis was then performed on each cluster using the FindAllMarkers function, with a log fold change threshold of 0.25, to identify differentially expressed genes (DEGs). The DEGs were subsequently used to identify cell types within each cluster, and the results were manually verified in accordance with a previously published study (Adams et al., 2020; Strunz et al., 2020).

### 2.3 Bulk RNA sequencing data processing

For the bulk RNA-seq datasets, we obtained the annotation information of the probes for these datasets, mapped them to their respective genes, removed instances of multiple matches, and calculated the median value as the gene expression for genes with multiple probes, and finally obtained the gene expression profile. DEGs were calculated using the limma package (version 3.46.0). DEGs were determined using the limma package (version 3.46.0) (Ritchie et al., 2015). Genes with an adjusted *p*-value <0.05 and an absolute logFC >0.5 were considered significantly dysregulated. Volcano and heatmap plots were generated using the ggplot2 package (version 3.3.5).

### 2.4 Gene ontology (GO) and KEGG pathway enrichment analysis

To identify the potential functional pathways of arterial endothelial cells in IPAH lungs, GO and KEGG enrichment analysis were applied using the R package clusterProfiler (version 4.6.2). Briefly, a gene expressed with |log2FC| >0.5 and adjust *p*-value < 0.05 between the IPAH group and the control group was determined to be a DEG. GO and KEGG enriched pathways were each applied by inputting DEGs into the clusterProfiler function enrichGO and enrichKEGG.

### 2.5 Gene set enrichment analysis (GSEA)

We identified dysregulation of predefined physiological pathways using the Gene Set Enrichment Analysis (GSEA v. 2.1.0) (Liberzon et al., 2011). The potential mechanisms were explored in the Molecular Signatures Database (MSigDB) of hallmarker gene sets (h.all.v2022.1.Hs. symbols). The significant results of GSEA were identified using the following statistical cutoff values: a nominal *p*-value < 0.05 and false discovery rate (FDR) < 0.25. AUC values for gene set enrichment were computed using the AUCell R package (version 1.6.1).

### 2.6 Protein-protein interaction (PPI) network analysis and hub gene screening

PPI analysis was used to examine the interaction co-expressed genes among DEGs from VE arteries cluster in GSE169471 dataset, DEGs from lungs in GSE113439 dataset, and DEGs from arterial endothelial cells in GSE185479 dataset. The STRING database was used to identify PPIs between co-expressed genes. PPI networks were visualized and analyzed using Cytoscape (version 3.8.0). The Hub genes were captured by the Cytoscape plug-in Molecular Complex Detection (MCODE) and cytoHubba.

### 2.7 Validation of hub genes expression

To avoid false positive rates, we validated all identified hub genes in GSE126262 and GSE130391. The comparison between the IPAH and control groups in both sets was conducted using a *t*-test, with a significance level of *p* < 0.05.

### 2.8 Animals and construction of PAH mouse model

All animal experiments were conducted using protocols approved by the Animal Care Committee of Jiangnan University. To induce PAH in C57BL/6J mice (Shanghai Laboratory Animal Co., Ltd., China), 6-week-old mice were subjected to a protocol involving a 3-week administration of weekly 20 mg/kg SU5416 injections while being exposed to chronic hypoxia (10% oxygen), as described previously (Rodor et al., 2022). Upon completion of the protocol, right ventricular systolic pressure (RVSP) was measured under terminal anesthesia (4% isoflurane), and the mice were euthanized by exsanguination.

### 2.9 Statistical analysis

In scRNA-seq analysis, the signature markers for each cluster were identified using the Wilcoxon rank-sum test to determine whether the expression of a specific class of genes was altered in IPAH lungs. The *p* values of GO and KEGG enrichment analysis were computed with a hypergeometric test and adjusted for multiple hypothesis testing with the Benjamini-Hochberg procedure. R studio software was used to draw graphics and conduct statistical analysis. *p* < 0.05 indicated statistical significance.

## 3 Results

**FIGURE 1:**
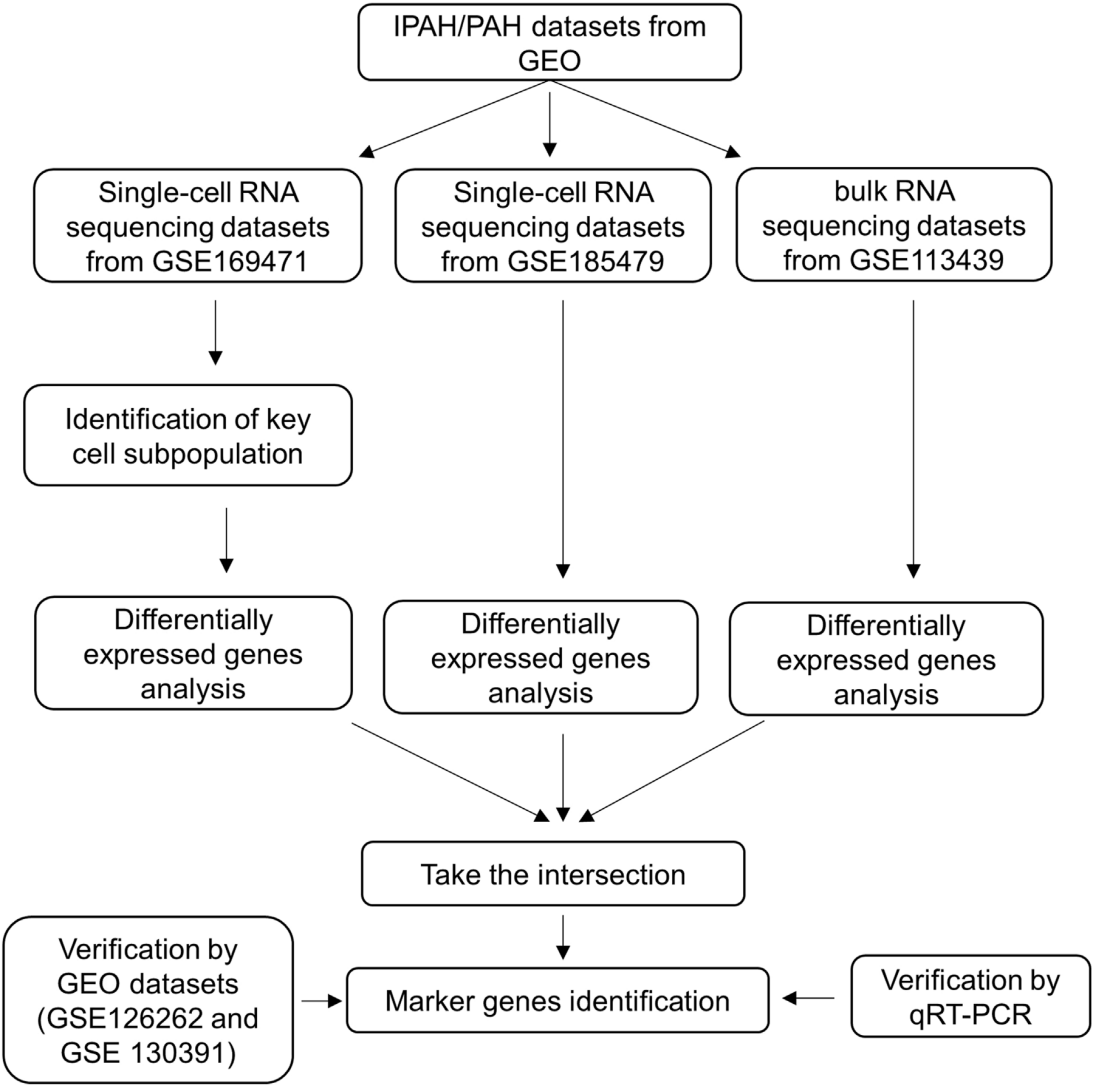
Flow chart of the research process.

The flow chart of our study is shown in Figure 1.

### 3.1 Single-cell RNA sequencing reveals cellular heterogeneity in IPAH

**FIGURE 2:**
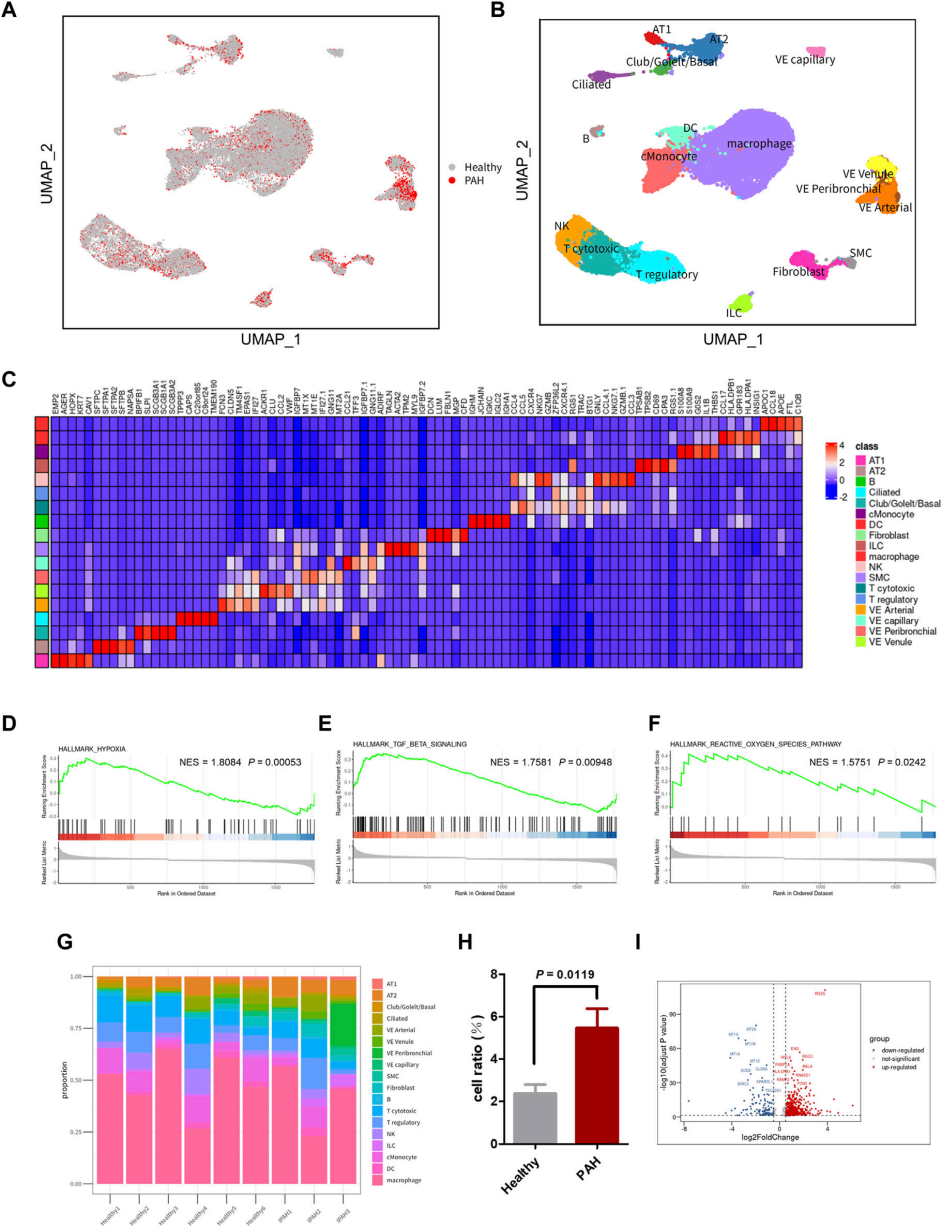
scRNA-seq reveals cellular heterogeneity in Healthy and PAH lungs. **(A)** UMAP plot of aggregate cells from IPAH and Healthy patients (after quality control, there were 10,454 cells from IPAH group and 19,731 cells from Healthy group). **(B)** UMAP visualization of clustering revealing 18 cell clusters. **(C)** Heatmap of conserved marker genes per cluster. AT1, Type I alveolar cells; AT2, Type Ⅱ alveolar cells; ciliated, ciliated airway epithelial cells; Club/Goblet/Basal, secretory club cells/Goblet cells/basal airway epithelial cells; SMC, smooth muscle cells; EC, endothelial cells; cMonocyte, classical monocytes; ILC, innate lymphoid cells; DC, dendritic cells; NK, natural killer cells; B, B cells; T cytotoxic, Cytotoxic T cell; T regulatory, T Regulatory Cells; VE Arterial, vascular arterial endothelial cells; VE Venule, vascular venule endothelial cells; VE Peribronchial, peribronchial vascular endothelial cells; VE capillary, capillary endothelial cells. **(D–F)** GSEA revealed hypoxia pathway **(D)**, TGF β signaling pathway **(E)**, and ROS pathway **(F)** were significantly upregulated in IPAH group, *p*-value <0.05, NES, normalized enrichment score. **(G)** Bar chart of the relative proportions of cell types from aggregated lung tissues. **(H)** Bar graph showed the cell proportion of VE Arterial cluster in Healthy group or IPAH group, *p*-value <0.05 vs. Healthy. **(I)** Volcano plot of DEGs in VE Arterial cluster from Healthy group and IPAH group.

The scRNA-seq dataset (GSE169471) from the GEO database was analyzed, consisting of 30,185 cells, including 10,454 cells from 3 IPAH lungs and 19,731 cells from 6 healthy control donor lungs (Figure 2A). After applying quality control and filtering cells based on the number of reported genes (see methods), individual transcriptional profiles of cells from IPAH lungs and healthy controls were included in the analysis (Supplementary Figures S1A, B). Unbiased clustering identified 18 discrete cell types based on distinct markers (Figure 2C), comprising 4 types of epithelial cells (AT1, AT2, Club/Goblet/Basal, and Ciliated), 6 types of stromal cells (Fibroblast, SMC, VE capillary, VE Venule, VE peribronchial, and VE arterial), 3 types of myeloid cells (cMonocyte, DC, and macrophage), and 5 types of lymphocytes (B, NK, T cytotoxic, T regulatory, and ILC), as shown by UMAP (Figure 2B).

To investigate the genetic signature changes in lung tissue affected by IPAH, we used the FindMarkers to identify genes exhibiting significant alterations. Subsequently, we conducted an enrichment analysis of biological pathways using the GSEA method, revealing the hypoxia pathway, TGF β signaling pathway, and reactive oxygen species pathway as the three pathways with the highest enrichment scores among the differentially expressed genes in lung tissues (Figures 2D–F; Supplementary Table S2). These findings suggest that identifying the cells involved in these pathways may help to reveal the pathogenesis of IPAH.

### 3.2 VE arteries involved in the progression of IPAH

**FIGURE 3:**
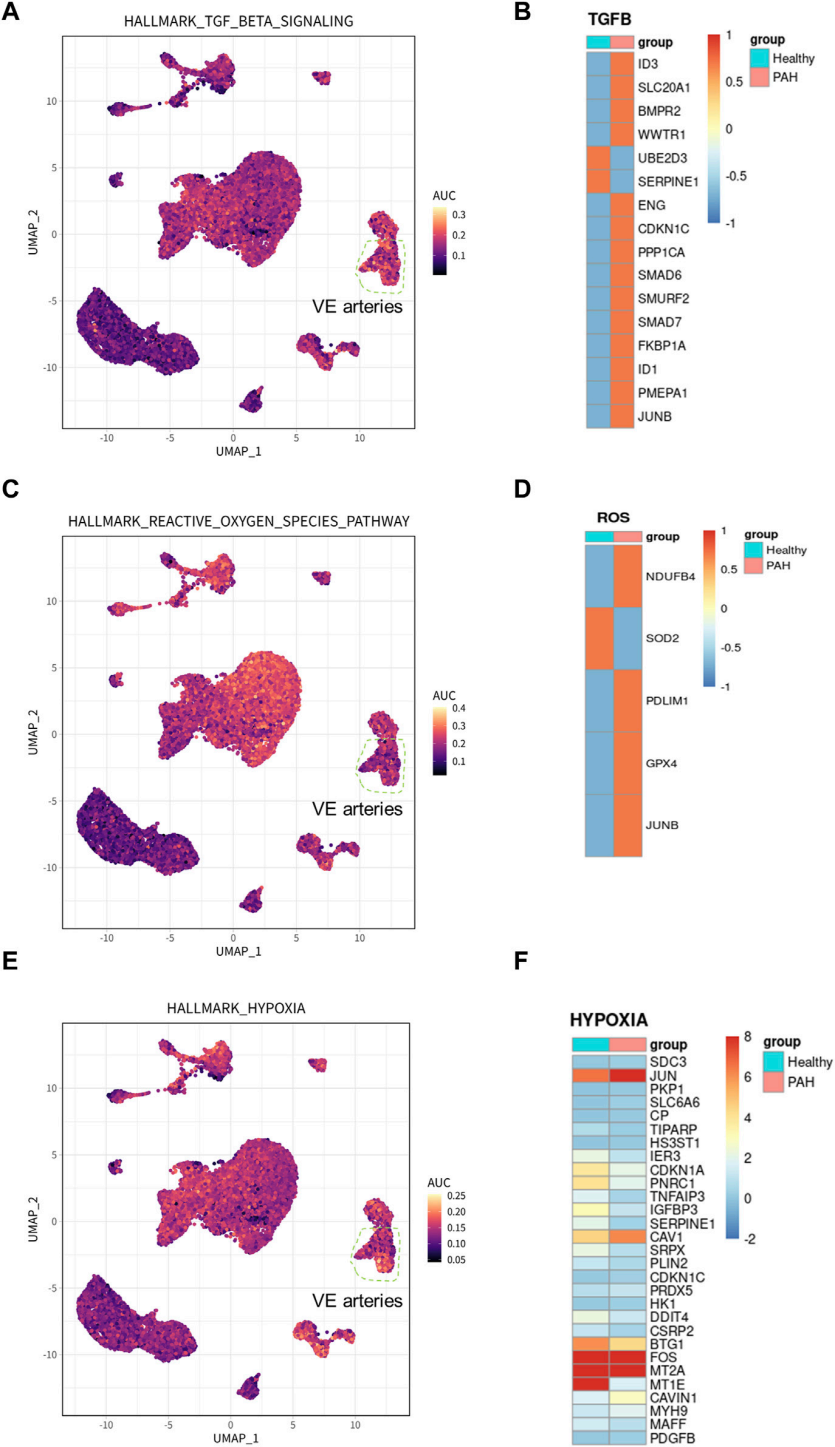
Hallmark pathway in arterial endothelial cells and related gene expression in IPAH. **(A)** UMAP plot showed genes involved in TGF β signaling pathway in all clusters. The genes were scored using AUCell. **(B)** Heatmap showed the expression of genes involved in TGF β signaling pathway in VE Arterial cluster. **(C)** UMAP plot showed genes involved in ROS pathway in all clusters. The genes were scored using AUCell. **(D)** Heatmap showed the expression of genes involved in ROS pathway in VE Arterial cluster. **(E)** UMAP plot showed genes involved in hypoxia pathway in all clusters. The genes were scored using AUCell. **(F)** Heatmap showed the expression of genes involved in hypoxia pathway in VE Arterial cluster.

To identify the cell types involved in idiopathic pulmonary arterial hypertension, we compared the proportion of each cell population in healthy lung tissue and IPAH lung tissue (Figure 2G). Our findings revealed that the proportion of arterial endothelial cell population was significantly higher in IPAH lung tissue compared to healthy lung tissue (Figure 1H; Supplementary Figure S2). Next, we used the AUCell R package to assess the activity of the hypoxic pathway, TGF β signaling pathway, and reactive oxygen species pathway in different cell types. Our analysis revealed that the VE arteries cluster had the highest AUC scores in the TGF β signaling pathway, while the macrophages cluster, epithelial clusters, and VE arteries cluster ranked highest in the reactive oxygen species pathway (Figures 3A, C). Moreover, VE arteries cluster and SMC cluster showed higher scores in the hypoxic pathway compared to other cell types analyzed (Figure 3E). These findings reveal that VE arteries cluster are involved in the progression of IPAH.

Since the VE artery cluster was significantly increased in the IPAH group, we proceeded to identify the DEGs within this cluster in IPAH (Figure 2I). Notably, RGS5 (log_2_FC = 2.63, *p* < 0.0001), a regulator of G-protein signaling that plays an important role in the inhibition of signal transduction, was strongly upregulated in IPAH. Consistent with this finding, previous reports have indicated that RGS5 expression was elevated in tumor-derived endothelial cells and barely detected in normal vascular system endothelial cells (Silini et al., 2012). This finding reveals a “tumorigenic” character of endothelial cells in idiopathic pulmonary arterial hypertension. We further performed GO biological process and KEGG pathway analysis of DEGs in VE arteries cluster, and found that these terms were mainly related to cell migration, cell adhesion, reactive oxygen species, and oxidative phosphorylation (Supplementary Figures S3A, B).

Additionally, DEGs from the VE arteries cluster were screened for genes involved in the hypoxic pathway, TGF β signaling pathway, and reactive oxygen pathway, demonstrating their expression in IPAH. The results demonstrated an increase in the expression of most DEGs involved in the TGF β signaling pathway (Figure 3B). Furthermore, the results indicated an increase in the expression of DEGs related to the reactive oxygen species pathway (NDUFB4, PDLIM1, GPX4, and JUNB), and a decrease in the expression of SOD, which has an antioxidant function, in the presence of idiopathic pulmonary hypertension (Figure 3D). In addition, among the DEGs involved in the hypoxic pathway in IPAH, the expression of CAV1, JUN, and PDGFB, which promote the progression of pulmonary hypertension, were all increased (Figure 3F). In conclusion, the results provide ample evidence that the VE arteries cluster is involved in the progression of IPAH.

### 3.3 DEGs of IPAH lung from bulk sequencing data

**FIGURE 4:**
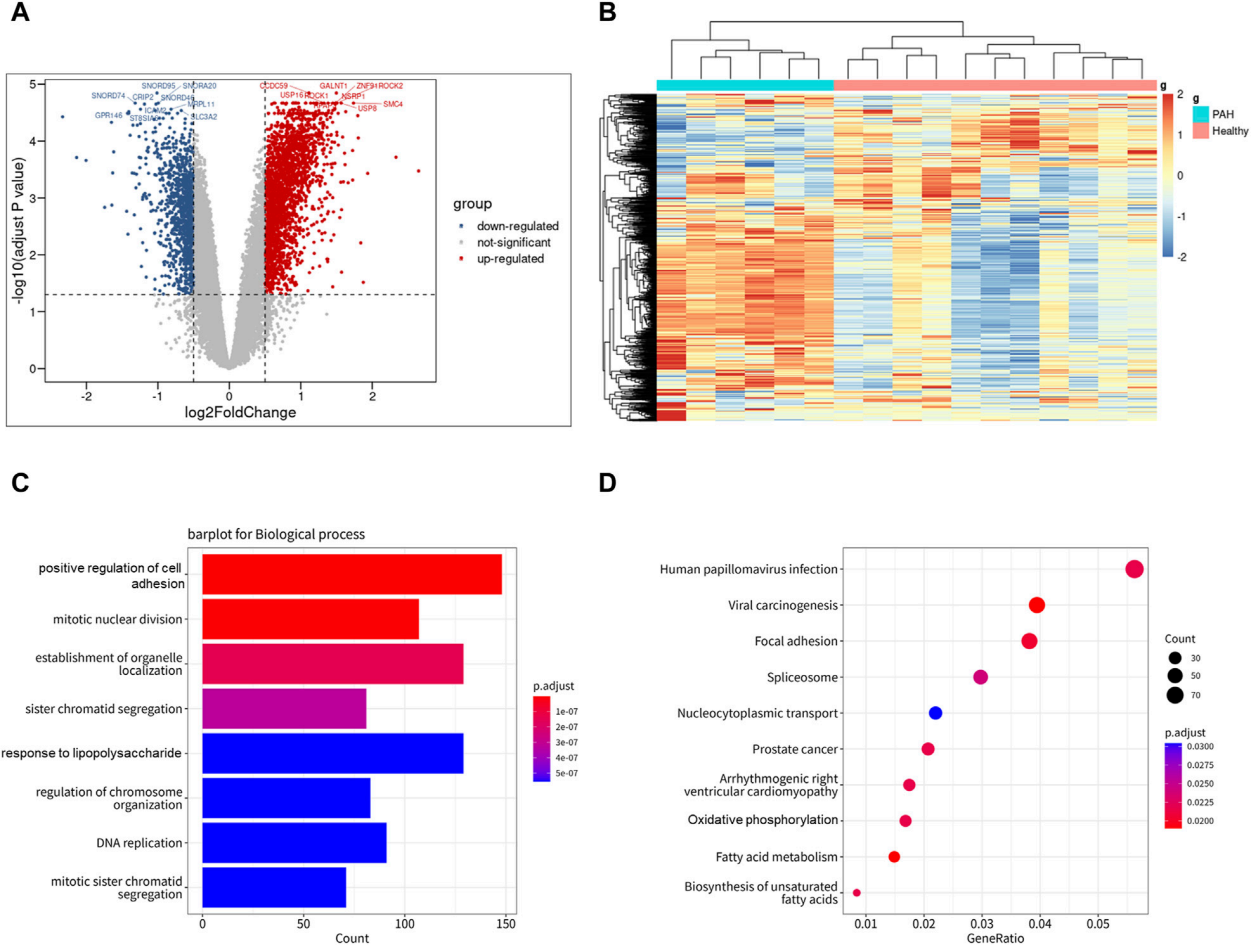
Differentially expressed genes of IPAH lungs from GSE113439 dataset. **(A)** Volcano plot of DEGs (|logFC| > 0.5 and adjusted *p*-value <0.05). Upregulated genes were colored in red and downregulated genes were colored in blue. **(B)** Heatmap of top 100 up and top 100 down DEGs of GSE113439. **(C)** GO biological process of DEGs in GSE113439. The top 8 GOs include positive regulation of cell adhesion, mitotic nuclear division, establishment of organelle localization, sister chromatid segregation, response to lipopolysaccharide, regulation of chromosome organization, DNA replication, mitotic sister chromatid segregation. **(D)** KEGG pathway of DEGs in GSE113439. The top 10 KEGGs include Pathways in Human papillomavirus infection, Viral carcinogenesis, Focal adhesion, Spliceosome, Nucleocytoplasmic transport, Prostate cancer, Arrhythmogenic right ventricular cardiomyopathy, Oxidative phosphorylation, Fatty acid metabolism, and Biosynthesis of unsaturated fatty acids.

To investigate the expression features of lung tissues in IPAH, the bulk RNA sequencing dataset GSE113439, which included 6 IPAH patients and 11 controls, was analyzed to explore DEGs in IPAH lungs. DEGs with adjusted *p* values <0.05, and |logFC| > 0.5 were selected. Thereafter, 2,340 upregulated and 1,125 downregulated DEGs were retained in IPAH lungs (Figure 4A). A heatmap of the top 100 upregulated and top 100 downregulated DEGs is shown, and relative consistency was observed within groups (Figure 4B). Interestingly, similar to the VE arteries cluster-enriched pathway in the lung of IPAH (Supplementary Figures S3A, B), the top 10 GO biological processes and KEGG pathways of DEGs in IPAH lungs also focused on cell adhesion and oxidative phosphorylation (Figures 4C, D). These data indicate that DEGs of the VE arteries cluster in IPAH share similar enriched pathways with DEGs in IPAH lungs, suggesting a potential role of arterial endothelial cells in the lung of IPAH patients.

### 3.4 PPI networks construction and hub genes identification

**FIGURE 5:**
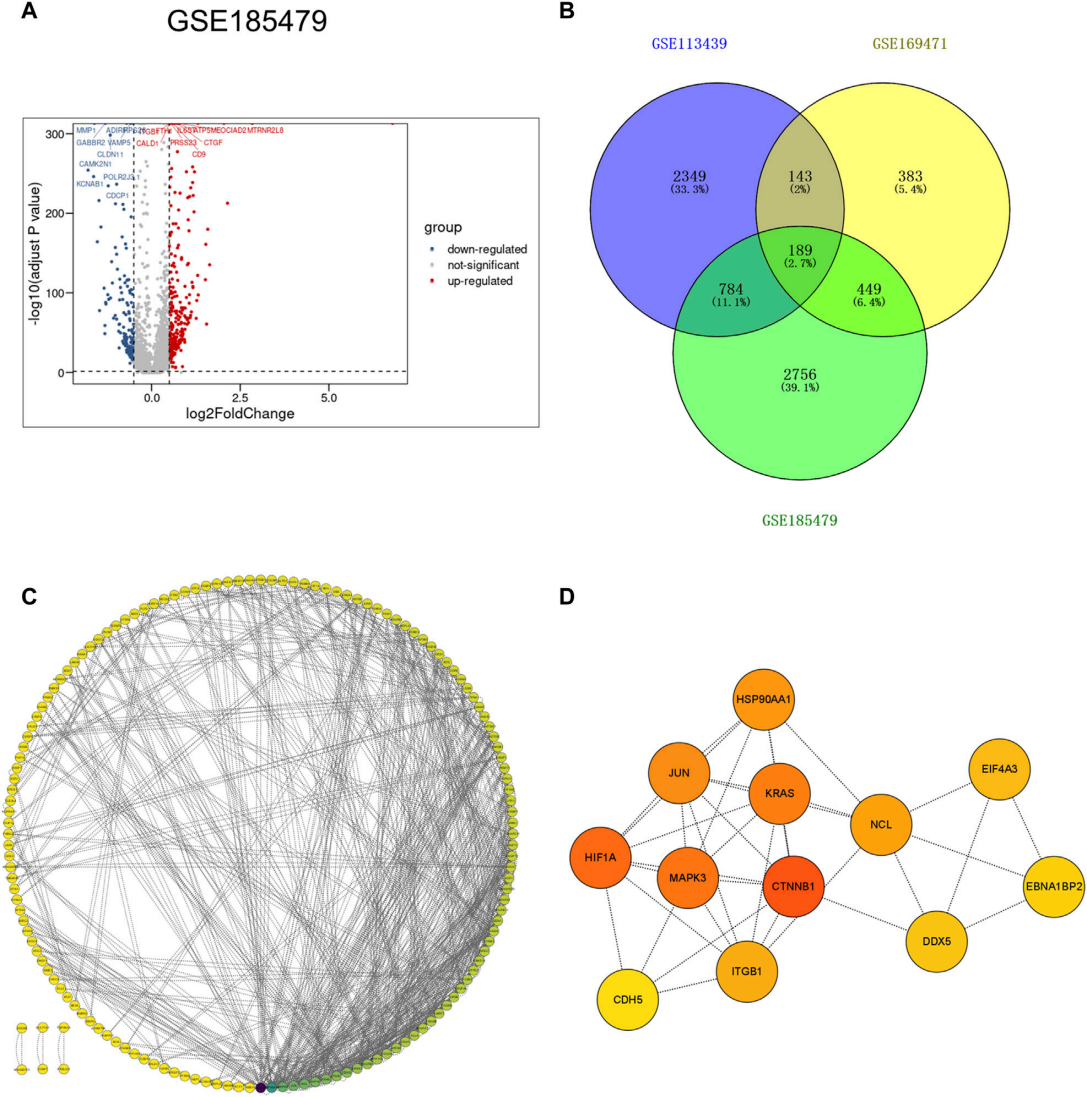
Identification of co-expressed differentially expressed genes and hub genes. **(A)** Volcano plot of DEGs (|logFC| > 0.2 and adjusted *p*-value <0.05) in GSE185479. Upregulated genes were colored in red and downregulated genes were colored in blue. **(B)** Venn plot showed co-expressed genes among DEGs of VE Arterial cluster in GSE169471, DEGs of IPAH lung in GES113439, and DEGs of arterial ECs in GSE185479. A total of 189 co-expressed genes were found. **(C)** The PPI network of the co-expressed genes illustrated using Cytoscape. **(D)** The hub genes were selected from the PPI network with CytoHubba plug-in in Cytoscape based on two topological methods (cytoHubba and MCODE). The darker the color, the higher the gene score.

To further explore gene expression changes in arterial endothelial cells in idiopathic pulmonary hypertension, the scRNA-seq dataset GSE185479, which included arterial endothelial cells from 3 PAH patients and 3 controls (Supplementary Figure S4), was analyzed to explore DEGs (Figure 5A). DEGs with adjusted *p* values <0.05, and |logFC| > 0.2 were selected. In total, 2,389 upregulated and 1,069 downregulated DEGs were retained in arterial endothelial cells in PAH. Moreover, we extracted the co-expressed genes among DEGs from the VE arteries cluster in the GSE169471 dataset, DEGs from lungs in the GSE113439 dataset, and DEGs from arterial endothelial cells in the GSE185479 dataset. The results showed 189 genes were screened as potential crosstalk genes by Venn diagrams (Figure 5B), indicating a common pathogenesis in IPAH.

To further study the interaction between common shared DEGs in IPAH, a PPI network of 189 DEGs was established based on the STRING database and visualized by Cytoscape software (Figure 5C). The hub genes were selected from the PPI network with CytoHubba plug-in in Cytoscape based on two topological methods (cytoHubba and MCODE). After intersection, twelve genes including CTNNB1, MAPK3, HIF1A, JUN, KRAS, HSP90AA1, ITGB1, CDH5, NCL, EIF4A3, DDX5, and EBNA1BP2, were found (Figure 5D), and their functional information is shown (Supplementary Table S4). Therefore, these genes could be considered as the kernel targets of IPAH.

### 3.5 Identification of diagnostic biomarkers

**FIGURE 6:**
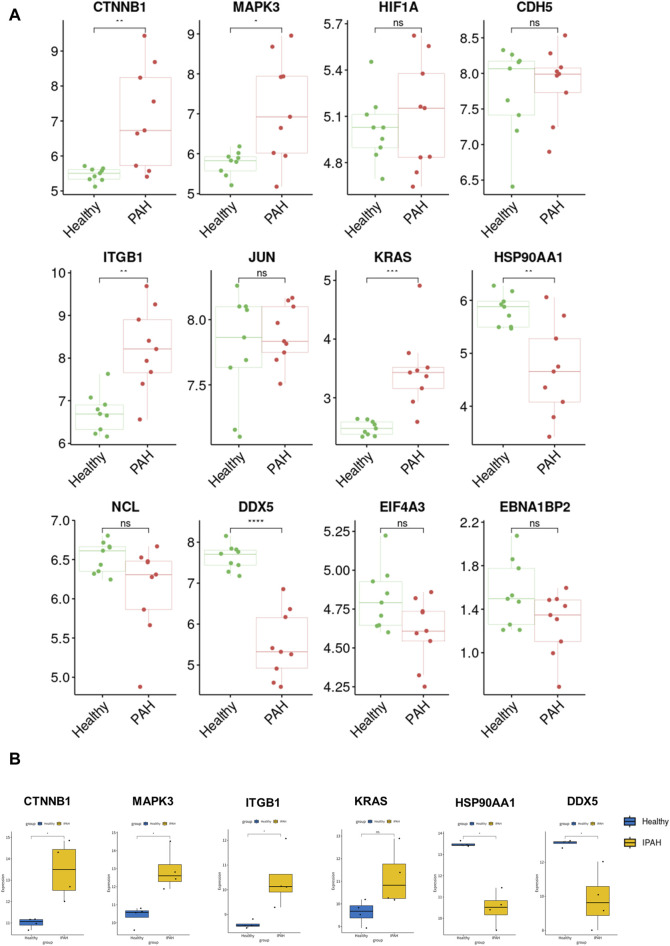
Validation of the identified hub genes in the PAH-related dataset. **(A)** The expression of CTNNB1, MAPK3, HIF1A, CDH5, ITGB1, JUN, KRAS, HSP90AA1, NCL, DDX5, EIF4A3, and EBNA1BP2 between Healthy and PAH groups in GSE126262 dataset. *p < 0.05, **p < 0.01, ***p < 0.001, ****p < 0.0001. **(B)** The expression of CTNNB1, MAPK3, ITGB1, KRAS, HSP90AA1, and DDX5 between Healthy and IPAH groups in GSE130391 dataset. *p < 0.05.

**FIGURE 7:**
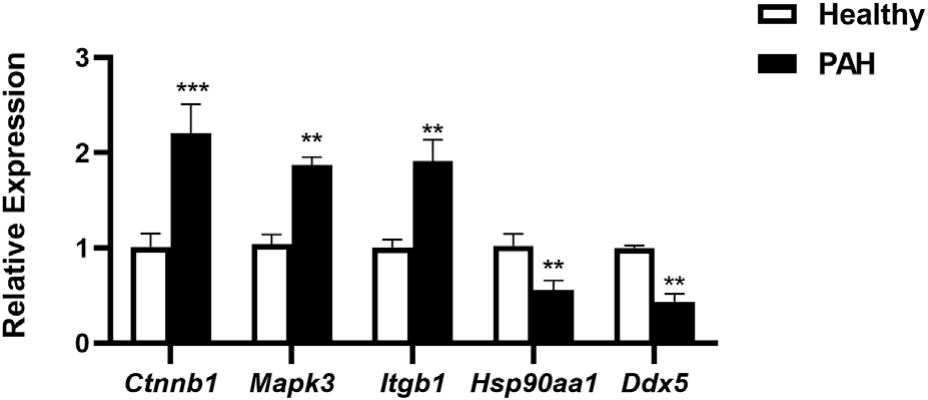
The mRNA expression of Ctnnb1, Mapk3, Itgb1, Hsp90aa1, and Ddx5 in pulmonary artery endothelial cells of Healthy and PAH mice. **p < 0.01, ***p < 0.001 by Student’s t-test.

For verification of the reliability of the 12 hub genes, we first validated the expression of these genes in the GSE126262 dataset, which contains IPAH-related arterial EC samples. We found that the expression of CTNNB1, MAPK3, ITGB1, and KRAS were significantly upregulated, while the expression of HSP90AA1 and DDX5 were significantly downregulated in arterial EC under IPAH compared to the healthy group (Figure 6A). To further confirm the expression of these six genes in lungs from IPAH patients, we then validated the expression of these genes in the GSE130391 dataset. The results showed that except for KRAS, the expression changes of the other 5 genes in the lung tissue of patients with IPAH were similar to those in the GSE126262 dataset (Figure 6B). Furthermore, our investigation involved the assessment of mRNA expression levels for five genes (Ctnnb1, Mapk3, Itgb1, Hsp90aa1, and Ddx5) within mouse pulmonary artery endothelial cells, conducted via RT-qPCR. The results demonstrated significant alterations in the mRNA levels of these five genes when comparing the PAH group to the Healthy group (Figure 7). Given the above, CTNNB1, MAPK3, ITGB1, HSP90AA1 and DDX5 might hold a powerful discrimination capability as potential biomarkers for IPAH disease.

## 4 Discussion

The dysfunctional EC signaling pathway tightly regulates multiple aspects of IPAH, including vascular tone, metabolism, remodeling and inflammation (Caruso et al., 2017; Gairhe et al., 2021). Given the mounting evidence highlighting the critical role of ECs in both the initiation and progression of IPAH, there is potential for developing novel therapies targeting multiple aspects of EC dysfunction and signaling, particularly the nodal signaling molecules regulating various pathways involved in the pathogenesis of IPAH (Condon et al., 2022; Rajagopal and Yu, 2022). Recent studies (Sangam et al., 2023; Walters et al., 2023) have unveiled that the dysregulation of SOX17 expression in IPAH patients significantly contributes to the disruption of endothelial functional homeostasis, thereby playing a pivotal role in the initiation and progression of pulmonary hypertension. Furthermore, it is noteworthy that SPARC, secreted by lung endothelial cells, functions as a paracrine factor, instigating pulmonary arterial smooth muscle cell (PASMC) proliferation and thereby facilitating the advancement of IPAH (Veith et al., 2022). Previous studies utilizing lung tissue transcriptomics have identified several biomarkers for the diagnosis of PAH, some of which are associated with endothelial cells (Pektaş et al., 2017; Marra et al., 2018; Didriksen et al., 2022). However, the heterogeneity and transcriptional features of ECs associated with IPAH remain largely unknown. In this study, we comprehensively characterized the cellular composition of the lungs and gained novel insights into altered gene expression profiles in IPAH vascular endothelial cells. Additionally, we identified biomarkers for the diagnosis of IPAH by integrating bulk RNA sequencing and single-cell RNA sequencing (scRNA-seq) data.

Initially, using scRNA-seq data, we illustrated the landscape of lung cells and gene characteristics, particularly focusing on arterial ECs in IPAH. We identified a total of 18 clusters, with an increased proportion of arterial endothelial cells found in the IPAH group compared to the Healthy group. Furthermore, utilizing AUCell analysis, we revealed that arterial endothelial cells are implicated in IPAH-enriched signaling pathways, including the hypoxia pathway, TGF β signaling pathway, and reactive oxygen species pathway. In the bulk RNA sequencing data from lungs of IPAH patients, we identified 2,340 upregulated and 1,125 downregulated differentially expressed genes (DEGs). GO analysis and KEGG pathway analysis of the bulk RNA sequencing data shared enrichment pathways with scRNA-seq data from lung tissue, further corroborating the involvement of arterial endothelial cells in IPAH progression. Collectively, these findings suggest that arterial endothelial cells play a key role in the progression of IPAH.

Subsequently, we delved deeper into the molecular mechanisms associated with arterial endothelial cells in triggering IPAH. Through a protein-protein interaction analysis of three pulmonary hypertension datasets, we identified 189 co-expressed genes and confirmed 12 potential biomarkers for the diagnosis of IPAH. To validate these potential biomarkers, we performed individual gene characterization on the arterial endothelial cell bulk RNA sequencing dataset of PAH and the lung dataset of IPAH, respectively. The results demonstrated that six of these biomarkers can be considered reliable biomarkers for IPAH. Furthermore, it is noteworthy that BMPR2 mutations in patients with pulmonary artery involvement also exert a significant influence on the development of PAH. Previous studies have reported (Wang et al., 2023) that PASMCs in PAH patients with BMPR2 mutations exhibit CTNNB1 nuclear translocation, indicating a potential interaction between BMPR2 and the markers identified in this study. MAPK3, one of the identified biomarkers, is expressed in both ECs and SMCs of pulmonary artery, and when activated, it promotes cell proliferation and aggravates the progression of PAH (Tao et al., 2019). ITGB1 (Integrin Subunit Beta 1) is a protein coding gene, plays a role in the biological functions of macrophages in lung tissue, such as stabilizing cell migration and promoting NO production, and reducing its expression may play a therapeutic role in PAH (Chen et al., 2018). HSP90AA1, another candidate biomarker for identification, has previously been shown (He et al., 2023) to be strongly associated with the increase of infiltrating T cells in PAH and serves as a potential analytical target for the identification of PAH. DDX5, a member of the RNA helicase family, plays a role in the regulation of ATP-dependent RNA helicase activities and cellular function, and is also a possible target molecule for the treatment of PAH (Hang et al., 2024). Naturally, the confirmation of the identified biomarker molecules through immunohistochemical experiments would enhance the credibility and reliability of the data derived from this study.

In conclusion, our study highlights the potential involvement of arterial endothelial cells in the progression of IPAH and elucidates genetic characteristic changes in IPAH through bioinformatic analysis combining both scRNA and bulk sequencing data. Moreover, we identified CTNNB1, MAPK3, ITGB1, HSP90AA1, and DDX5 as promising biomarkers for the diagnosis of IPAH disease.

## Data Availability

The datasets presented in this study can be found in online repositories. The names of the repository/repositories and accession number(s) can be found in the article/Supplementary Material.

## References

[B1] Adams T. S. Schupp J. C. Poli S. Ayaub E. A. Neumark N. Ahangari F. (2020). Single-cell rna-seq reveals ectopic and aberrant lung-resident cell populations in idiopathic pulmonary fibrosis. Sci. Adv. 6 (28), eaba1983. 10.1126/sciadv.aba1983 32832599 PMC7439502

[B2] Asosingh K. Comhair S. Mavrakis L. Xu W. Horton D. Taylor I. (2021). Single-cell transcriptomic profile of human pulmonary artery endothelial cells in health and pulmonary arterial hypertension. Sci. Rep. 11 (1), 14714. 10.1038/s41598-021-94163-y 34282213 PMC8289993

[B3] Caruso P. Dunmore B. J. Schlosser K. Schoors S. Dos Santos C. Perez-Iratxeta C. (2017). Identification of microrna-124 as a major regulator of enhanced endothelial cell glycolysis in pulmonary arterial hypertension via Ptbp1 (polypyrimidine tract binding protein) and pyruvate kinase M2. Circulation 136 (25), 2451–2467. 10.1161/circulationaha.117.028034 28971999 PMC5736425

[B4] Cella G. Bellotto F. Tona F. Sbarai A. Mazzaro G. Motta G. (2001). Plasma markers of endothelial dysfunction in pulmonary hypertension. Chest 120 (4), 1226–1230. 10.1378/chest.120.4.1226 11591565

[B5] Chen H. Y. Pan L. Yang H. L. Xia P. Yu W. C. Tang W. Q. (2018). Integrin Alpha5beta1 suppresses rbmscs anoikis and promotes nitric oxide production. Biomed. Pharmacother. = Biomedecine Pharmacother. 99, 1–8. 10.1016/j.biopha.2018.01.038 29324307

[B6] Condon D. F. Agarwal S. Chakraborty A. Auer N. Vazquez R. Patel H. (2022). Novel mechanisms targeted by drug trials in pulmonary arterial hypertension. Chest 161 (4), 1060–1072. 10.1016/j.chest.2021.10.010 34655569 PMC9005865

[B7] Didriksen H. Molberg Ø. Mehta A. Jordan S. Palchevskiy V. Fretheim H. (2022). Target organ expression and biomarker characterization of chemokine Ccl21 in systemic sclerosis associated pulmonary arterial hypertension. Front. Immunol. 13, 991743. 10.3389/fimmu.2022.991743 36211384 PMC9541617

[B8] Eichstaedt C. A. Song J. Benjamin N. Harutyunova S. Fischer C. Grünig E. (2016). Eif2ak4 mutation as "second hit" in hereditary pulmonary arterial hypertension. Respir. Res. 17 (1), 141. 10.1186/s12931-016-0457-x 27809840 PMC5095976

[B9] Evans C. E. Cober N. D. Dai Z. Stewart D. J. Zhao Y. Y. (2021). Endothelial cells in the pathogenesis of pulmonary arterial hypertension. Eur. Respir. J. 58 (3), 2003957. 10.1183/13993003.03957-2020 33509961 PMC8316496

[B10] Farber H. W. Miller D. P. Poms A. D. Badesch D. B. Frost A. E. Muros-Le Rouzic E. (2015). Five-year outcomes of patients enrolled in the reveal registry. Chest 148 (4), 1043–1054. 10.1378/chest.15-0300 26066077

[B11] Gairhe S. Awad K. S. Dougherty E. J. Ferreyra G. A. Wang S. Yu Z. X. (2021). Type I interferon activation and endothelial dysfunction in caveolin-1 insufficiency-associated pulmonary arterial hypertension. Proc. Natl. Acad. Sci. U. S. A. 118 (11), e2010206118. 10.1073/pnas.2010206118 33836561 PMC7980434

[B12] Girerd B. Lau E. Montani D. Humbert M. (2017). Genetics of pulmonary hypertension in the clinic. Curr. Opin. Pulm. Med. 23 (5), 386–391. 10.1097/mcp.0000000000000414 28661905

[B13] Halliday S. J. Matthews D. T. Talati M. H. Austin E. D. Su Y. R. Absi T. S. (2020). A multifaceted investigation into molecular associations of chronic thromboembolic pulmonary hypertension pathogenesis. JRSM Cardiovasc. Dis. 9, 2048004020906994. 10.1177/2048004020906994 32110389 PMC7019411

[B14] Hang C. Zu L. Luo X. Wang Y. Yan L. Zhang Z. (2024). Ddx5 targeted epigenetic modification of pericytes in pulmonary hypertension following intrauterine growth restriction. Am. J. Respir. Cell Mol. Biol. 10.1165/rcmb.2023-0244OC 38301267

[B15] He X. Fang J. Gong M. Zhang J. Xie R. Zhao D. (2023). Identification of immune-associated signatures and potential therapeutic targets for pulmonary arterial hypertension. J. Cell. Mol. Med. 27 (23), 3864–3877. 10.1111/jcmm.17962 37753829 PMC10718157

[B16] Hughes R. Tong J. Oates C. Lordan J. Corris P. A. (2005). Evidence for systemic endothelial dysfunction in patients and first-order relatives with pulmonary arterial hypertension. Chest 128 (6 Suppl. l), 617s. 10.1378/chest.128.6_suppl.617S 16373872

[B17] Jiang Q. Liu C. Liu S. Lu W. Li Y. Luo X. (2021). Dysregulation of bmp9/bmpr2/smad signalling pathway contributes to pulmonary fibrosis and pulmonary hypertension induced by bleomycin in rats. Br. J. Pharmacol. 178 (1), 203–216. 10.1111/bph.15285 33080042

[B18] Korsunsky I. Millard N. Fan J. Slowikowski K. Zhang F. Wei K. (2019). Fast, sensitive and accurate integration of single-cell data with Harmony. Nat. methods 16 (12), 1289–1296. 10.1038/s41592-019-0619-0 31740819 PMC6884693

[B19] Legchenko E. Chouvarine P. Borchert P. Fernandez-Gonzalez A. Snay E. Meier M. (2018). PPARγ agonist pioglitazone reverses pulmonary hypertension and prevents right heart failure via fatty acid oxidation. Sci. Transl. Med. 10 (438), eaao0303. 10.1126/scitranslmed.aao0303 29695452

[B20] Marra A. M. Bossone E. Salzano A. D'Assante R. Monaco F. Ferrara F. (2018). Biomarkers in pulmonary hypertension. Heart Fail. Clin. 14 (3), 393–402. 10.1016/j.hfc.2018.03.005 29966636

[B21] Mura M. Cecchini M. J. Joseph M. Granton J. T. (2019). Osteopontin lung gene expression is a marker of disease severity in pulmonary arterial hypertension. Respirol. Carlt. Vic. 24 (11), 1104–1110. 10.1111/resp.13557 30963672

[B22] Naeije R. Richter M. J. Rubin L. J. (2022). The physiological basis of pulmonary arterial hypertension. Eur. Respir. J. 59 (6), 2102334. 10.1183/13993003.02334-2021 34737219 PMC9203839

[B23] Pektaş A. Olguntürk R. Kula S. Çilsal E. Oğuz A. D. Tunaoğlu F. S. (2017). Biomarker and shear stress in secondary pediatric pulmonary hypertension. Turkish J. Med. Sci. 47 (6), 1854–1860. 10.3906/sag-1609-13 29306249

[B24] Rajagopal S. Yu Y. A. (2022). The pathobiology of pulmonary arterial hypertension. Cardiol. Clin. 40 (1), 1–12. 10.1016/j.ccl.2021.08.001 34809910

[B25] Reyes-Palomares A. Gu M. Grubert F. Berest I. Sa S. Kasowski M. (2020). Remodeling of active endothelial enhancers is associated with aberrant gene-regulatory networks in pulmonary arterial hypertension. Nat. Commun. 11 (1), 1673. 10.1038/s41467-020-15463-x 32245974 PMC7125148

[B26] Ritchie M. E. Phipson B. Wu D. Hu Y. Law C. W. Shi W. (2015). Limma powers differential expression analyses for rna-sequencing and microarray studies. Nucleic acids Res. 43 (7), e47. 10.1093/nar/gkv007 25605792 PMC4402510

[B27] Rodor J. Chen S. H. Scanlon J. P. Monteiro J. P. Caudrillier A. Sweta S. (2022). Single-cell rna sequencing profiling of mouse endothelial cells in response to pulmonary arterial hypertension. Cardiovasc. Res. 118 (11), 2519–2534. 10.1093/cvr/cvab296 34528097 PMC9400412

[B28] Runo J. R. Loyd J. E. (2003). Primary pulmonary hypertension. Lancet London, Engl. 361 (9368), 1533–1544. 10.1016/s0140-6736(03)13167-4 12737878

[B29] Sangam S. Sun X. Schwantes-An T. H. Yegambaram M. Lu Q. Shi Y. (2023). Sox17 deficiency mediates pulmonary hypertension: at the crossroads of sex, metabolism, and genetics. Am. J. Respir. Crit. care Med. 207 (8), 1055–1069. 10.1164/rccm.202203-0450OC 36913491 PMC10112457

[B30] Saygin D. Tabib T. Bittar H. E. T. Valenzi E. Sembrat J. Chan S. Y. (2020). Transcriptional profiling of lung cell populations in idiopathic pulmonary arterial hypertension. Pulm. Circ. 10 (1). 10.1177/2045894020908782 PMC7052475 32166015

[B31] Silini A. Ghilardi C. Figini S. Sangalli F. Fruscio R. Dahse R. (2012). Regulator of G-protein signaling 5 (Rgs5) protein: a novel marker of cancer vasculature elicited and sustained by the tumor's proangiogenic microenvironment. Cell. Mol. life Sci. CMLS 69 (7), 1167–1178. 10.1007/s00018-011-0862-8 22130514 PMC3299962

[B32] Strunz M. Simon L. M. Ansari M. Kathiriya J. J. Angelidis I. Mayr C. H. (2020). Alveolar regeneration through a Krt8+ transitional stem cell state that persists in human lung fibrosis. Nat. Commun. 11 (1), 3559. 10.1038/s41467-020-17358-3 32678092 PMC7366678

[B33] Tao W. Sun W. Zhu H. Zhang J. (2019). Mir-205-5p suppresses pulmonary vascular smooth muscle cell proliferation by targeting mical2-mediated erk1/2 signaling. Microvasc. Res. 124, 43–50. 10.1016/j.mvr.2019.03.001 30853343

[B34] Veith C. Varturk-Ozcan I. Wujak M. Hadzic S. Wu C. Y. Knoepp F. (2022). Sparc, a novel regulator of vascular cell function in pulmonary hypertension. Circulation 145 (12), 916–933. 10.1161/CIRCULATIONAHA.121.057001 35175782

[B35] Walters R. Vasilaki E. Aman J. Chen C. N. Wu Y. Liang O. D. (2023). Sox17 enhancer variants disrupt transcription factor binding and enhancer inactivity drives pulmonary hypertension. Circulation 147 (21), 1606–1621. 10.1161/CIRCULATIONAHA.122.061940 37066790 PMC7614572

[B36] Wang L. Moonen J. R. Cao A. Isobe S. Li C. G. Tojais N. F. (2023). Dysregulated smooth muscle cell bmpr2-arrb2 Axis causes pulmonary hypertension. Circulation Res. 132 (5), 545–564. 10.1161/CIRCRESAHA.121.320541 36744494 PMC10008520

[B37] Wolff B. Lodziewski S. Bollmann T. Opitz C. F. Ewert R. (2007). Impaired peripheral endothelial function in severe idiopathic pulmonary hypertension correlates with the pulmonary vascular response to inhaled iloprost. Am. heart J. 153 (6), 1088.e1–e7. 10.1016/j.ahj.2007.03.005 17540215

