# Supplementary material for: Identification of potential biomarkers for idiopathic pulmonary arterial hypertension using single-cell and bulk RNA sequencing analysis
Source: Front Genet. 2024 Mar 22;15:1328234. doi: 10.3389/fgene.2024.1328234 (PMC10995363; doi:10.3389/fgene.2024.1328234)
Supplement: Supplementary file 1 [file DataSheet1.PDF]

Supplementary Material

Identification of biomarkers for the diagnosis of Idiopathic pulmonary arterial hypertension by integrating single-cell RNA sequencing and bulk RNA sequencing datasets

Supplemental Materials and Methods

Endothelial cell preparation and culture

Primary mouse pulmonary arterial endothelial cells (MPAECs) were isolated from Healthy and PAH mice and used for RT-qPCR. Briefly, lungs were dissection in PBS and incubated with type IA collagenase (2 mg/ml, sigma, USA) for 15min at 37°C. Detached endothelial cells were collected by centrifugation, resuspended in endothelial cell growth medium (EGM) supplemented with bovine brain extract (Lonza, USA) and culture in a flask at 37°C with 5% CO_2. Non-adherent cells were removed 2 h later. The endothelial cells were cultured in EGM at 37°C with 5% CO_2 for 1-2 days(1).

RNA extraction and RT-PCR

The total RNA was extracted from the mice in Healthy and PAH groups using the Trizol reagent (CWBIO, China). The concentration of RNA was measured by an ultraviolet spectrophotometer (NanoDrop one, USA). Reverse transcription was performed in a total volume of 20 μL. The total RNA (500 ng/μL, 2 μL) was mixed gDNA wiper Mix (4 μL, Vazyme, China) and RNase-free ddH_2O (10 μL), watered bath at 42°C for 2 min, and then added the reverse transcriptase mix (5x HiScript III qRT SuperMix, 4μL, Vazyme). Reverse transcription reaction was performed at 37°C for 15min, 85°C for 5s and 4°C for 5min. Quantitative PCR for cDNAs was conducted using the SYBR Mix reagent (Vazyme, China) and 7500 Real time PCR system, as previously described(2). Primer sequences (GENEWIZ, China) for related genes were used (Table. S4).

Supplemental figures and tables

1.1 Supplementary Figures

Supplementary Figure 1. Quality control of single-cell RNA data in GSE169471. (A) The genes (features), counts, mitochondrial gene percentage, ribosome gene percentage, and erythrocyte of each sample. (B) PCs selection using JackStraw function.

Supplementary Figure 2. Graph demonstrates the proportion of each cell type in health and IPAH. Refer to methods for statistical tests used.

Supplementary Figure 3. GO and KEGG pathway analysis of DEGs in VE Arterial cluster. (A) Histogram of the top 8 GO biological process in VE Arterial cluster. (B) Bubble chart of the top 10 KEGG pathway in VE Arterial cluster.

Supplementary Figure 4. scRNA analysis of arterial endothelial cells in GSE185479. (A) UMAP plot of arterial endothelial cells from PAH and Healthy patients. (B) UMAP visualization of clustering revealing 13 cell clusters. (C) UMAP plot of integrated all cells from GSE185479 and GSE169471. (D) UMAP visualization of clustering revealing VE arterial subpopulation. (E) Feature plot of PECAM1, CDH5, CLDN5, TM4SF1.

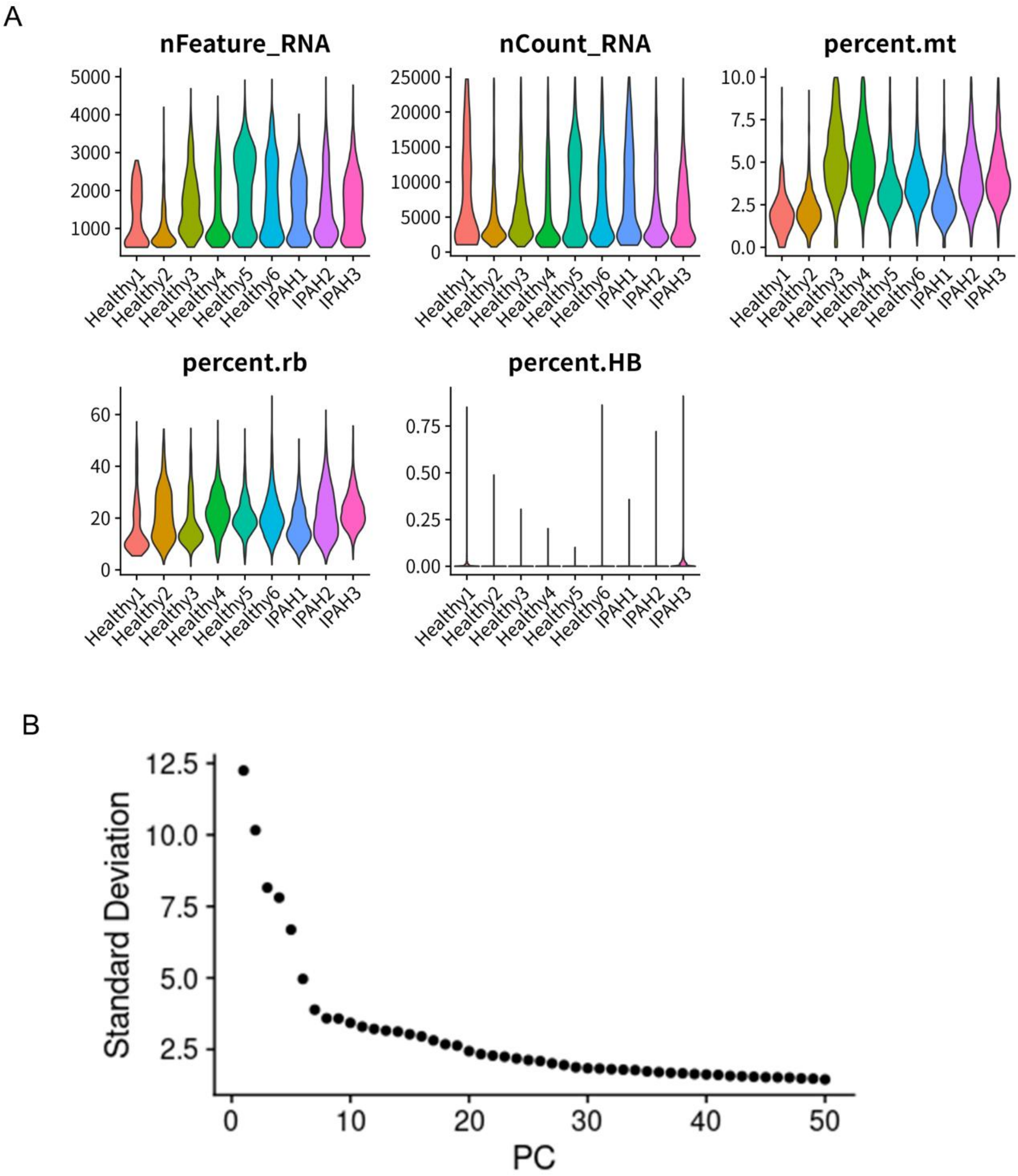
A
nFeature_RNA
nCount_RNA
percent.mt
percent.rb
percent.HB
Healthy1
Healthy2
Healthy3
Healthy4
Healthy5
Healthy6
IPAH1
IPAH2
IPAH3
B
Standard Deviation
PC

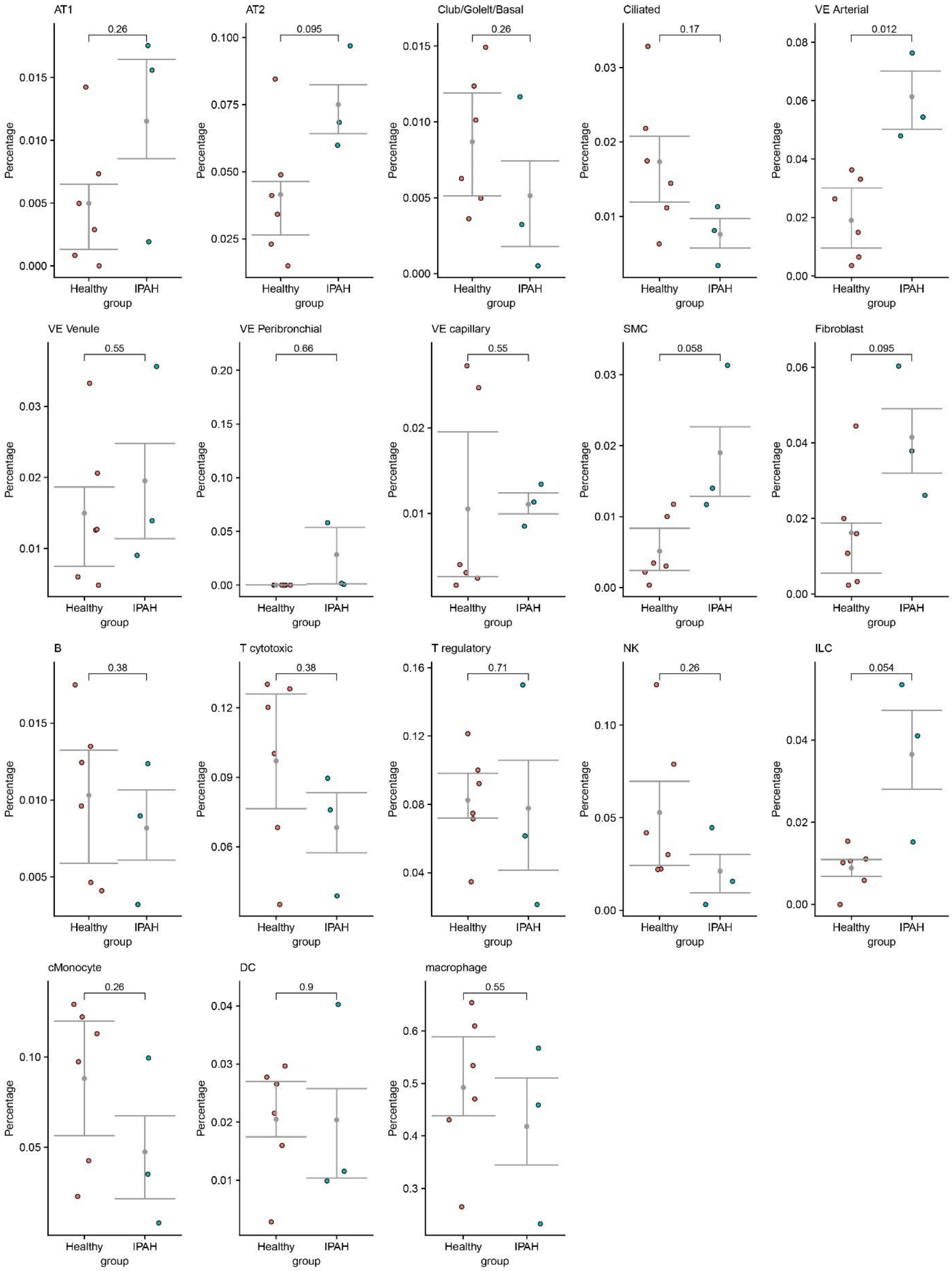
AT1
0.26
AT2
0.095
Club/Golelt/Basal
0.26
Ciliated
0.17
VE Arterial
0.012
VE Venule
0.55
VE Peribronchial
0.66
VE capillary
0.55
SMC
0.058
Fibroblast
0.095
B
0.38
T cytotoxic
0.38
T regulatory
0.71
NK
0.26
ILC
0.054
cMonocyte
0.26
DC
0.9
macrophage
0.55
Percentage
Healthy
IPAH
group

A

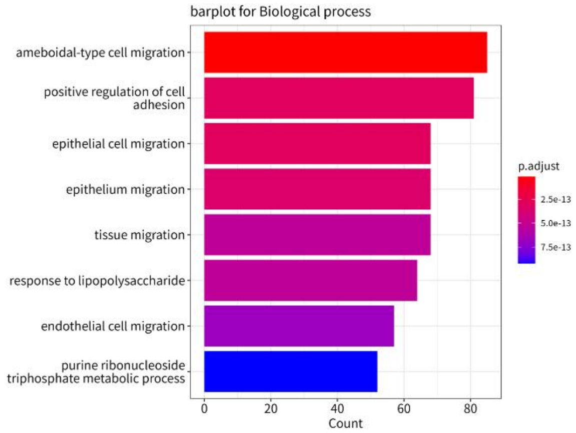

barplot for Biological process
ameboidal-type cell migration
positive regulation of cell adhesion
epithelial cell migration
epithelium migration
tissue migration
response to lipopolysaccharide
endothelial cell migration
purine ribonucleoside triphosphate metabolic process
0
20
40
60
80
Count
p.adjust
2.5e-13
5.0e-13
7.5e-13

B

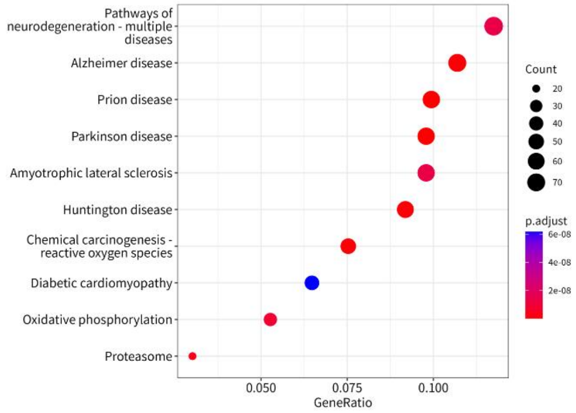

Pathways of neurodegeneration - multiple diseases
Alzheimer disease
Prion disease
Parkinson disease
Amyotrophic lateral sclerosis
Huntington disease
Chemical carcinogenesis - reactive oxygen species
Diabetic cardiomyopathy
Oxidative phosphorylation
Proteasome
0.050
0.075
0.100
GeneRatio
Count
20
30
40
50
60
70
p.adjust
6e-08
4e-08
2e-08

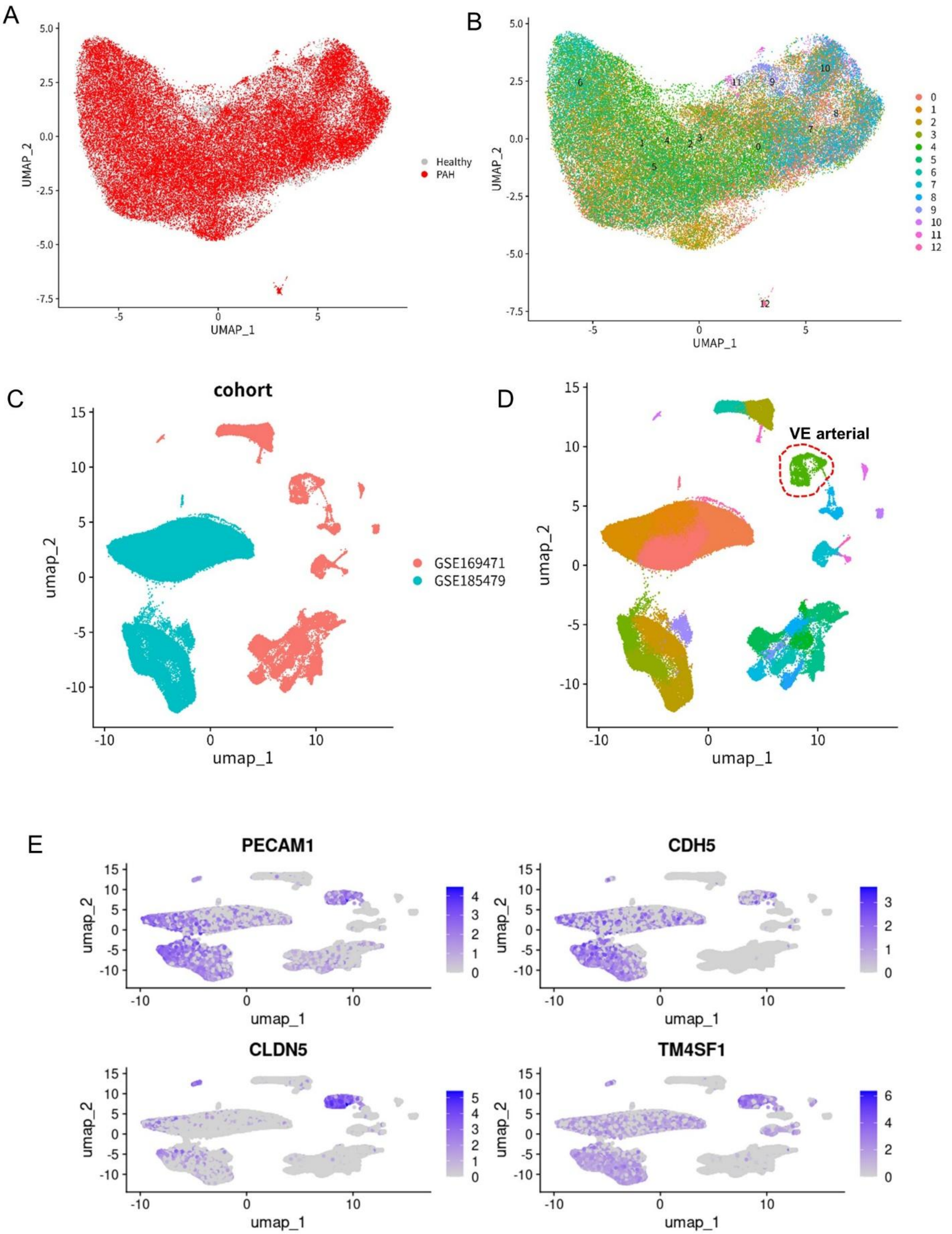
A
UMAP_1
UMAP_2
Healthy
PAH
B
UMAP_1
UMAP_2
0
1
2
3
4
5
6
7
8
9
10
11
12
C
cohort
umap_1
umap_2
GSE169471
GSE185479
D
VE arterial
umap_1
umap_2
E
PECAM1
CDH5
CLDN5
TM4SF1
umap_1
umap_2

1.2 Supplementary Tables

Supplementary Table 1. Summary of datasets used in this study.

Accession ID	Disease	Year	Data type	Tissue type	Controls/Cases
GSE169471	IPAH	2021	Single-cell RNA	lung	6/3
GSE185479	PAH	2021	Single-cell RNA	lung endothelial cells	3/3
GSE113493	IPAH	2018	Bulk-RNA	lung	11/6
GSE126262	PAH	2019	Bulk-RNA	lung endothelial cells	9/9
GSE130391	IPAH	2019	Bulk-RNA	lung	4/4

Supplementary Table 2. GSEA of top 10 pathways in IPAH.

Pathway	NES	pvalue
HALLMARK_HYPOXIA	1.808396656	0.000537572
HALLMARK_TGF_BETA_SIGNALING	1.758163973	0.009483883
HALLMARK_REACTIVE_OXYGEN_SPECIES_PATHWAY	1.575106025	0.024205805
HALLMARK_EPITHELIAL_MESENCHYMAL_TRANSITION	1.565872147	0.053418803
HALLMARK_MYC_TARGETS_V2	1.528530922	0.070981211
HALLMARK_UNFOLDED_PROTEIN_RESPONSE	1.478313816	0.066239316
HALLMARK_INFLAMMATORY_RESPONSE	1.466398648	0.053608247
HALLMARK_ESTROGEN_RESPONSE_EARLY	1.420552313	0.089912281
HALLMARK_GLYCOLYSIS	1.418720516	0.102345416
HALLMARK_TNFA_SIGNALING_VIA_NFKB	1.402416885	0.088842975

Supplementary Table 3. Functional roles of hub genes.

Gene Symbol	Protein	Function
CTNNB1	Catenin beta-1	Key downstream component of the canonical Wnt signaling pathway
MAPK3	Mitogen-activated protein kinase 3	Serine/threonine kinase which acts as an essential component of the MAP kinase signal transduction pathway
HIF1A	Hypoxia-inducible factor 1-alpha	Functions as a master transcriptional regulator of the adaptive response to hypoxia
JUN	Transcription factor AP-1	Transcription factor that recognizes and binds to the enhancer heptamer motif 5'-TGA[CG]TCA-3'
KRAS	GTPase KRas	Ras proteins bind GDP/GTP and possess intrinsic GTPase activity
HSP90AA1	Heat shock protein HSP 90-alpha	Molecular chaperone that promotes the maturation, structural maintenance and proper regulation of specific target proteins involved for instance in cell cycle control and signal transduction
ITGB1	Integrin beta-1	Integrins alpha-1/beta-1, alpha-2/beta-1, alpha-10/beta- 1 and alpha-11/beta-1 are receptors for collagen
CDH5	Cadherin-5	Cadherins are calcium-dependent cell adhesion proteins
NCL	Nucleolin	Nucleolin is the major nucleolar protein of growing eukaryotic cells
EIF4A3	Eukaryotic initiation factor	Core component of the splicing-dependent multiprotein exon junction complex (EJC) deposited at splice junctions on mRNAs

	4A-III	
DDX5	Probable ATP-dependent RNA helicase DDX5	Involved in the alternative regulation of pre-mRNA splicing
EBNA1BP2	Probable rRNA-processing protein EBP2	Required for the processing of the 27S pre-rRNA

Supplementary Table 4. primer sequence

Gene name	Forward	Reverse
Ctnnb1	ATGGAGCCGGACAGAAAAGC	TGGGAGGTGTCAACATCTTCTT
Mapk3	TCCGCCATGAGAATGTTATAGGC	GGTGGTGTTGATAAGCAGATTGG
Itgb1	ATGCCAAATCTTGCGGAGAAT	TTTGCTGCGATTGGTGACATT
Hsp90aa1	GACGCTCTGGATAAAATCCGTT	TGGGAATGAGATTGATGTGCAG
Ddx5	TTCCTGCGAATGTCATGGATG	TCCAGTCTGAGCTACTCCAAC

Supplemental references

1. Fehrenbach ML, Cao G, Williams JT, Finklestein JM, Delisser HM. Isolation of Murine Lung Endothelial Cells. *American journal of physiology Lung cellular and molecular physiology* (2009) 296(6):L1096-103. Epub 2009/03/24. doi: 10.1152/ajplung.90613.2008.

2. Feng B, Yao PM, Li Y, Devlin CM, Zhang D, Harding HP, et al. The Endoplasmic Reticulum Is the Site of Cholesterol-Induced Cytotoxicity in Macrophages. *Nature cell biology* (2003) 5(9):781-92. Epub 2003/08/09. doi: 10.1038/ncb1035.
